# Unveiling the mechanisms of synthetic compounds against *Candida auris*: An integrative review

**DOI:** 10.1016/j.crphar.2025.100231

**Published:** 2025-08-08

**Authors:** Yamini Saini, Zeeshan Fatima, Muriel Billamboz, Saif Hameed

**Affiliations:** a Amity Institute of Biotechnology, Amity University Haryana, Manesar, Gurugram, 122413, India; b ICL, JUNIA, Université Catholique de Lille, LITL, F-59000, Lille, France

**Keywords:** *Candida auris*, Synthetic compounds, Biofilm, Cell wall, efflux pump, Cell organelle

## Abstract

The multidrug-resistant fungal species *Candida auris* has drawn attention from across the world due to its capacity to elude traditional therapies and flourish in medical environments. Its resilience, which includes biofilm development and efflux-mediated drug resistance, highlighted the need for novel antifungal approaches. Despite advancements in antifungal therapeutics, the rising prevalence of resistance and limited antifungal arsenal demand ongoing research into novel and more effective treatments. To tackle this rising issue, the available literature suggests several approaches. Among those, the use of synthetic compounds (SCs) appears as first-line option. However, to prove the efficacy of these SCs against *C. auris* a complete coverage is still elusive in a single study. Thus, in this integrative review, we aimed to summarize the anti-*C. auris* SCs that are reported in literature. About 47 articles were included in this review using predefined selection criteria. Data were extracted for detailed reviews from PubMed, Google scholar and Science direct. All the included studies tested antifungal activities of the SCs and evaluated their mode of actions. These data highlighted diverse modes of action such as perturbation of biofilm formation, disruption of cell wall and organelles, inhibition of efflux and generation of reactive oxygen species to name few. Taken together, SCs represent viable candidates for effective antifungal treatment. The information gathered in the present study emphasizes the need for further investigations, including preclinical studies and clinical trials, to evaluate the therapeutic potential of these agents against *C. auris*.

## Highlights

• This review gathers all updated data on synthetic compounds active against *Candida auris*.
• More than 1.200 reports have been screened to select 47 highly representative ones.
• Compounds are classified both by their mode of action and their chemical structures.
• The review will help medicinal chemists and biologists to develop new first-in-class antifungals against *C. auris*.

## Graphical abstract

**Figure:**
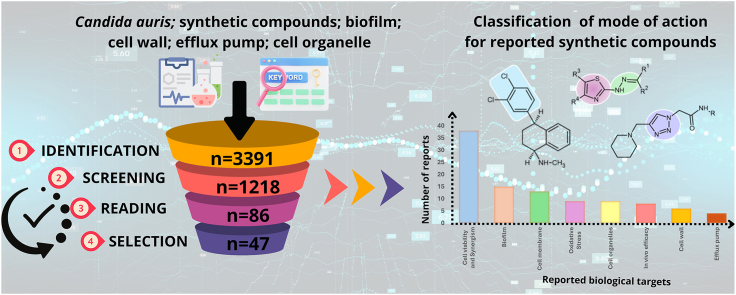

## 1 Introduction

Since its discovery in 2009 in Japan from a patient's ear canal (Satoh et al., 2009), *Candida auris*, a multidrug-resistant fungus species, has grown to pose a serious threat to world health. Its rapid spread and resistance to multiple antifungal treatments have earned it the label of an “emerging antibiotic-resistant pathogen”. Therefore, serious concerns raised among international health organizations (Das et al., 2023). This fungus is becoming increasingly associated with serious infections, particularly in hospital settings. Outbreaks in intensive care units have surged. The issue has been further exacerbated by the COVID 19 pandemic, as overwhelmed healthcare systems have facilitated its spread. *C. auris* belongs to a group of multidrug-resistant fungi and is classified into four main clades e.g. South Asian, East Asian, African, and South American. These clades exhibit distinct genetic and geographical characteristics, which complicates complicating diagnosis and treatment (De Luca et al., 2022). Approximately 1.5 million people die from invasive fungal diseases, including *C. auris* infections, each year, highlighting the urgent need for more effective treatments, better diagnostic tools, and enhanced infection prevention strategies (Puumala et al., 2022; Amann et al., 2023; Huang et al., 2024). According to CDC, there has been a concerning rise in instances of an antifungal-resistant pathogen that mostly spreads in healthcare settings and poses serious hazards to susceptible people. The number of cases in the United States has increased dramatically from 329 in 2018 to over 1000 in 2021, with notable outbreaks occurring in Southern California. Clusters of the disease have also been reported in China, India, Pakistan, and Southeast Asia, demonstrating its global reach ((Centers for Disease Control and Prevention, 2021); World Health Organization, 2020). Such an increase in cases underscored the urgent need to stop its spread and safeguard vulnerable groups by improving infection control procedures and creating novel treatments (Mohammad et al., 2019; Tetz et al., 2019; Ramli et al., 2024). As infectious diseases within organs with contagious damage continue to spread and pose serious threats to the medical community, antimicrobial resistance has emerged as a major worldwide threat. In response, the World Health Organization (WHO) developed a plan in 2015 to address this rising challenge (Levshin et al., 2024; Malūkaitė et al., 2021).

The rapid increase of multidrug-resistant bacteria has outpaced the standard drug discovery process for developing new antifungal agents. In response to this urgent need, scientists are exploring different approaches to identify antifungals treatments for *C. auris*. One such approach is the use of synthetic compounds to prevent or combat these Candida infections (Billamboz et al., 2021). To maximize the safety, efficacy and stability of synthetic compounds such as antibiotics, antifungals, and painkillers, they are chemically engineered to target these organisms (Gowri et al., 2020). When it comes to drug design, synthetic compounds outperform natural compounds based on several criteria. One of the key advantages is the ability to exert full control over the structure and properties of the designed compounds, eliminating any variability between batches that can be attributed to variations in plant or animal sources, environmental factors, and harvesting conditions. These are issues that are often encountered in the extraction of natural compounds. SCs can be precisely tailored to target specific biological mechanisms. Chemists can modify the molecular structure to achieve efficacy and selectivity and subsequently reduce side-effects. The SCs are fully customisable, which is particularly interesting for modulating stability, pharmacokinetics, bioavailability and the fate of the drug. Another advantage is the high scalability and cost-effectiveness of SCs. Unlike natural compounds, which are generally obtained through long, complex extraction processes, SCs are synthetized with mastered purity and quality in large -quantities. This process is generally more cost-effective and scalable for industrial production. Finally, the intellectual property and patentability are important considerations for the companies involved in drug discovery. Natural compounds, due to their natural origin, may be difficult to patent. The ability to create synthetic molecules de novo provides an opportunity for companies to protect their discoveries and recover research and development costs. Taken together, all these key features explain why SCs are currently being engineered as novel anti-Candida agents to fight against *C. auris*.

In response to the urgent need for practical solutions to the global burden of fungal diseases, this review identifies synthetic compounds as promising candidates for antifungal therapy. To confirm the potential of these compounds in therapeutic contexts, further research and clinical investigation are required. While numerous studies have demonstrated the therapeutic potential of synthetic compounds against *C. auris*, a review integrating all antifungal mechanisms elucidated in various studies is still lacking. This study highlights significant discoveries from 2009 to 2024 regarding the modes of action of synthetic antimicrobial drugs against multidrug-resistant *C. auris* and offers a thorough summary of the most recent developments in the field. Understanding these modes of action is crucial for optimizing targeted therapeutic interventions.

## 2 Literature survey

A detailed literature survey was conducted in accordance with the PRISMA (Protocol for Reporting Items for Systematic Reviews and Meta-Analysis) guidelines to evaluate the antifungal efficacy of synthetic agents against *C. auris* infections. Data were gathered from multiple databases including PubMed, Google Scholar, and ScienceDirect, using the following keywords: “*Candida auris* and synthetic compounds”, “*Candida auris* and synthetic drugs”, “*Candida auris* and synthetic products”, “*Candida auris* and synthetic molecules”, “*Candida auris* and synthetic chemicals”, “*Candida auris* and synthetic derivatives”. The selection process involved an initial screening of titles and abstracts, followed by a thorough review of full texts of the articles. Only studies addressing the antifungal effects of synthetic agents on *C. auris* in the English language were eligible for inclusion, while the rest were excluded from the final analysis. As this was a comprehensive review of the literature on scientific research, no statistical analysis was performed.

**Fig. 1:**
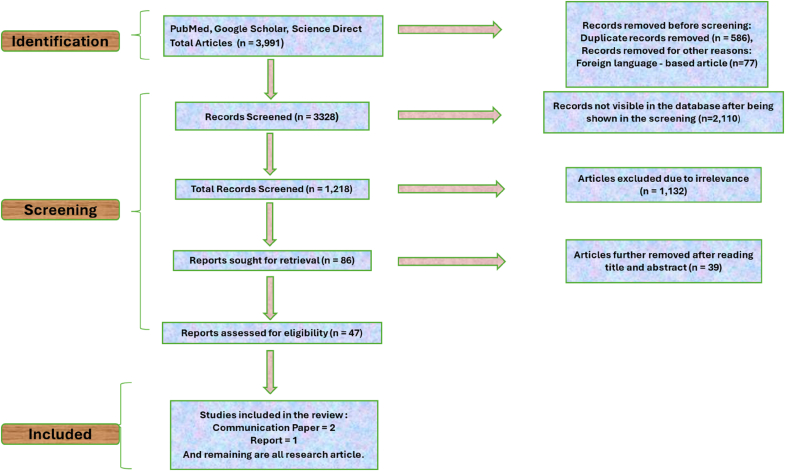
PRISMA Flowchart of literature search and screening strategy for the included studies (n = 47).

This review analysed a total of 3991 articles retrieved from PubMed, Google Scholar, and ScienceDirect. The aim was to refine this extensive pool of research and focus on studies providing high-quality insights into the topic to achieve a comprehensive understanding. The process began with the identification of these articles. Duplicate records (n = 586) and articles written in languages other than English (n = 77) were removed, resulting in 3328 articles being considered. During the screening stage, 2110 articles were excluded due to unavailability in the database. The remaining 1218 studies were reviewed for relevance by analysing their titles and abstracts. This led to the exclusion of a further 1132 articles that were not specific to *C. auris* but to other species. A total of 86 articles were then retrieved for detailed review. After careful evaluation, a further 39 studies were excluded due to insufficient alignment, reporting only *C. auris* inhibition screening and lacking further validation. Other reasons for exclusion included showing a high MIC, missing mode of action or therapeutic potential, and not aligned with the research focus. This left 47 studies that met the eligibility criteria and were included in the final selection (see Fig. 1). These comprised two communication papers, one report, and the remainder original research articles that directly addressed the central topic. The selected studies provided valuable insights and emphasised important themes relevant to the research focus. This methodical approach ensured that only relevant and high-quality studies were included in the analysis. Furthermore, the review identified areas of in the existing literature that require further research, highlighting gaps that warrant exploration.

## 3 Mechanisms of action for SCs in *C. auris*

**Fig. 2:**
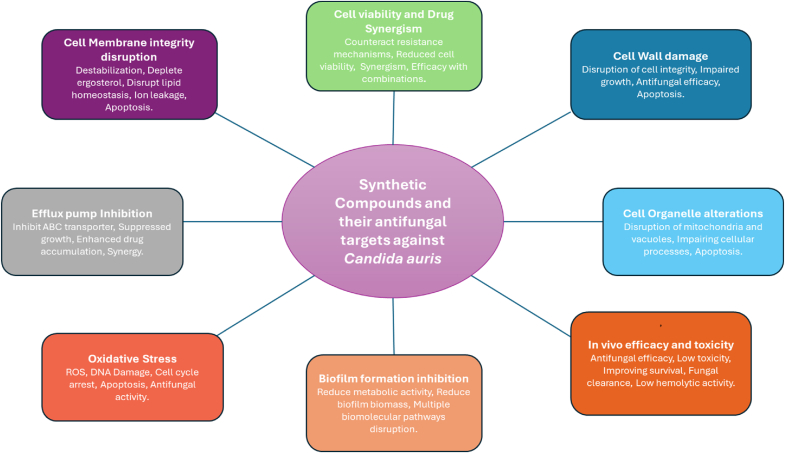
Diverse antifungal targets of SCs reported in the included studies (n = 47) for *C. auris.*

The SCs reported in the literature (Table 1) exert their antifungal effects through a diverse range of mechanisms. The following sections will summarize the various antifungal modes of action including several primary targets (Fig. 2).

**Table 1:** Characteristics of the included studies (n = 47).

S.No.	Author	Journal	Country	Name of SCs	Method of Analysis	Mechanism of action against *C. auris*
1	Mohammad et al. (2019)	Scientific Reports	USA	Compound 1 (synthetic phenylthiazole molecule)	1) Time Kill Assay, 2) Crystal Violet Reporter Assay, 3) XTT Reduction Assay, 4) Kinetic Microplate Reader	1) Compound 1 inhibited all eight strains of C. auris at 2 μg/mL, Fluconazole was largely ineffective. 2) Compound 1 showed rapid fungicidal activity against C. albicans and C. auris, like amphotericin B. 3) Compound 1 and amphotericin B inhibited C. auris biofilms effectively at 1∗ MIC but less at sub-MIC levels. 4) Compound 1 and amphotericin B reduced C. auris biofilm metabolic activity, with stronger effects at higher MICs. 5) Compound 1 significantly prolonged survival of C. auris-infected nematodes, like 5-Fluorocytosine.
2	Fatima et al. (2024)	Bioorganic Chemistry	India	**C4** (butyl 2-(4-chlorophenyl)hydrazine-1-carboxylate) and **C13** (phenyl 2-(4-chlorophenyl) hydrazine-1-carboxylate)	1) Broth Dilution Assay, 2) Spot Assay, 3) Static/Cidal Determination Assay, 4) Drug Synergism, 5) Cell Viability Assay, 6) R6G Extracellular Efflux Assay, 7) Haemolytic Activity Assay	1) C4 and C13, particularly C13, showed promising antifungal activity and low predicted toxicity. 2) C4 displayed fungicidal activity, while C13 was fungistatic, both reduced C. auris viability significantly. 3) C4 and C13 altered cell wall components and vacuole morphology, reducing acidification in C. auris. 4) C4 and C13 disrupt cell membrane integrity, deplete ergosterol, and inhibit ABC transporter function in C. auris. 5) C4 and C13 impaired C. auris growth on non-fermentative carbon sources, indicating mitochondrial dysfunction. 6) C4 and C13 induced oxidative stress in C. auris, causing lipid peroxidation and DNA damage, reversible by antioxidants. 7) C4 and C13 significantly reduced C. auris biofilm formation, metabolic activity, biomass, and epithelial adherence. 8) C4 and C13 improved C. elegans survival against C. auris with minimal host toxicity and low hemolytic activity.
3	(Malūkaitė et al., 2021)	Molecules	Lithuania	Compounds 2b and 5a (Thiazole Derivatives)	1) MTT Assay 2) Time Kill Assay 3) Biofilm Fomation Assay	1) Compounds 2b and 5a showed selective antifungal activity, with 4-ClC6H4 critical against C. auris.
4	Tetz et al. (2019)	Antimicrobial Agents and Chemotherapy	USA	MYC-053	1) Broth Microdilution Method 2) In vitro P. carinii and P. murina ATP assays	1) MYC-053 showed promising activity against C. auris with precise IC50 and MIC values.
5	Chu et al. (2021)	Antimicrobial Agents and Chemotherapy	USA	SCY-247 (analog of ibrexafungerp)	1) MIC Assay 2) MFC Assay	1) SCY-247 displayed potent activity against C. auris, with superior fungicidal effects compared to IBX. 2) SCY-247 exhibited time-kill efficacy against C. albicans, C. auris, S. apiospermum, and A. fumigatus.
6	Lee et al. (2021)	Pharmaceutics	South Korea	NAIMS 7c (Naphthalenyl-Acyl-Imidazolium Salts 7c)	1) Antifungal Susceptibility Microdilution Assay 2) Cell Viability Assay 3) Fungal Cell Growth Test 4) ROS detection cell-based assay kit 5) Quantitative Reverse Transcriptase PCR (qRT-PCR) analysis	1) NAIMS 7c demonstrated strong antifungal activity against C. auris, with a MIC of 3.125 μg/mL.
7	Levshin et al. (2024)	Pharmaceutics	Russia	**31a (L-173)**, (hybrid derivative of triazole and thiazolidine-2,4-dione)	1) MICs determined via RPMI microdilution. 2) Susceptibility tests were performed in triplicate.	1) Compound L-173 exhibited MIC values of 2 mg/L for *C. auris*, surpassing Voriconazole's 8 mg/L, respectively.
8	Amann et al. (2023)	International Journal of Molecular Sciences	Germany	**Cm-p5 Dimeric Derivatives** (parallel CysCysCm-p5 (Dimer 1) and anti parallel CysCysCm-p5 (Dimer 2))	1) Cell viability was measured using a resazurin assay method. 2) Crystal Violet Assay (for Biofilm Quantification) 3) Tecan Infinite F200 reader (to quantify biofilm biomass)	1) Dimer 1 and Dimer 2 showed moderate planktonic activity but effective anti-biofilm properties against C. auris. 2) Strains M1 and M2 showed increased fluconazole resistance compared to the parental C. auris strain. 3) Dimer 1 and Dimer 2 reduced biofilm growth of C. auris strains M1 (IC50 23.2 μg/mL) and M2 (IC50 33.3 μg/mL).
9	Kubiczek et al. (2020)	Antibiotics	Germany	**Cyclic Cm-p5, Dimer 1** and **Dimer 2**	1) Crystal Violet Assay 2) Absorbance Readings	1) Cm-p5 dimers significantly inhibited mature C. auris biofilm growth, achieving up to 95 %
10	Huang et al. (2024)	mSphere	South Korea	Liposomal **Fba** and **Met6** peptide	1) Murine immunization and serum analysis 2) ELISA assay 3) Enzyme-linked immunosorbent spot assay 4) Statistical analysis	1) F-SNAP and M-SNAP vaccines induced specific IgG responses against Fba and Met6 peptides related to C. auris. 2) SNAP vaccines showed stronger IgG responses and improved survival against C. auris compared to MP12. 3) Peptide-SNAP vaccines provided better protection against C. auris, showing higher survival and reduced fungal burden**.** 4) Peptide SNAP vaccines elicited robust IgG responses, reducing kidney CFU and supporting protective immunity against invasive candidiasis.
11	Srivastava et al. (2020)	Journal Of Advanced Research	South Africa	Piperidine based 1,2,3-triazolylacetamide derivatives **(pta1-pta6**)	1) Antifungal susceptibility testing 2) MUSE Cell Analyzer 3) Fluorescence Microscopy 4) Apoptotic assays 5) Mitochondrial membrane depolarization assay 6) Terminal deoxynucleotidyl transferase dUTP nick-end labelling (TUNEL) assay 7) Statistical analysis	1) Among six derivatives, pta1, pta2, and pta3 showed strong anti-Candida auris activity, with MICs of 0.24–0.97 μg/mL, outperforming AmB's 0.125–4 mg/mL. 2) MUSE viability assay shows pta1, pta2, pta3 dose-dependently reduce C. auris viability, supporting antimycotic efficacy. 3) pta1, pta2, pta3 disrupt C. auris plasma membrane integrity, triggering apoptosis-linked membrane destabilization and DNA impairment. 4) pta1, pta2, and pta3 induce mitochondrial depolarization in C. auris, reducing membrane potential and triggering apoptosis. 5) pta3, pta2, and pta1 induce C. auris apoptosis by cytochrome *c* release, disrupting mitochondrial function and activating caspases. 6) Test compounds, especially pta3, induced C. auris apoptosis, disrupting membrane integrity and externalizing phosphatidylserine at higher concentrations. 7) TUNEL assay confirmed test compounds induced dose-dependent DNA fragmentation and apoptosis in C. auris. 8) Piperidine compounds induce DNA damage, cell cycle arrest, and apoptosis in C. auris.
12	Hafidi et al. (2022)	Journal of Biomolecular Structure And Dynamics	Brazil	Synthesized cationic surfactants **CnEtOH** and **CnPrOH**	1) In vitro antifungal susceptibility test analysis (MIC and MFC)	1) C14EtOH shows lower MIC values against C. auris compared to C14PrOH and C16PrOH.
13	Hashemi et al. (2018)	Journal of Antimicrobial Chemotherapy	USA	Ceragenins	1) Broth Microdilution Assay 2) XTT Assay 3) Scanning Electron Microscopy Analysis 4) Confocal laser scanning microscopy 5) Ex Vivo Antifungal Assay	1) CSA-131 shows consistent antifungal activity across C. auris clades, including resistant strains. 2) CSA-131 disrupts C. auris cell membranes, causing morphological changes at higher concentrations. 3) C. auris biofilms show reduced susceptibility, especially to fluconazole, compared to C. albicans. 4) CSA-131 penetrates biofilms of C. auris and C**.** albicans**,** killing cells without altering biofilm structure**.** 5) Ceragenin gels and creams significantly reduce C. auris and C. albicans counts, outperforming nystatin and Monistat 7**.**
14	Liu et al. (2022)	PLoS Pathogens	China	**PLA-HA-aPDT (**nanofibrous membrane)	1) Photodynamic inactivation (PDI) assay 2) Agar plate dilution method 3) confocal laser scanning microscopy 4) Transmission electronic microscopy (TEM) 5) In vitro cytotoxicity assay 6) Hemolysis assay 7) XTT reduction Assay 8) UV–visible absorption spectrometer 9) Annexin V-FITC apoptosis detection kit 10) Mitochondrial membrane potential assay 11) Fluorescence microscopy 12) Metacaspase activation assay	1) PLA-HA with illumination shows potent, repeatable antifungal activity against C. auris, with <0.1 % survival. 2) Red fluorescence in PLA-HA-treated C. auris indicates cell wall damage, unlike intact walls in controls. 3) PLA-HA with illumination damages C. auris cells; slight L929 toxicity and excellent biocompatibility observed. 4) PLA-HA aPDT effectively eradicates C. auris biofilms, reducing viability by 77 % with illumination. 5) PLA-HA aPDT enhances wound healing in C. auris-infected rats with minimal inflammation and side effects. 6) PLA-HA aPDT induces apoptosis in C. auris via ROS production and mitochondrial damage.
15	Liu et al. (2022)	Drug Resistance Update	China	**COP1T-HA** (Cage-modified hypocrellin)	1) Antifungal susceptibility test 2) UV-2600 recording spectrophotometer 3) Agar plate dilution method 4) Confocal laser scanning microscopy 5) Cytotoxicity assays 6) Cell migration assays	1) COP1T-HA under laser effectively inhibits C. auris, causing mild cell wall damage. 2) COP1T-HA aPDT effectively eradicates C. auris biofilms, causing cell lysis and structural damage. 3) COP1T-HA shows promise for antifungal and wound healing applications, effectively targeting C. auris.
16	Puumala et al. (2022)	mBio	Canada	Trisubstituted isoxazole **MMV688766**	1) Drug susceptibility assays 2) Spectrophotometer 3) Mammalian cytotoxicity assays 4) C. elegans infection assays 5) Haploinsufficiency profiling 6) Lipid droplet assays 7) Reverse-phase LC-MS for nonpolar lipids 8) Ion-paired reverse-phase LC-MS for polar lipids. 9) FAS purification and activity assay 10) Blue native PAGE 11) Quantitative RT-PCR.	1) MMV688766 exhibits fungicidal activity against C. auris, inhibiting growth by 80 % at 25 mM 2) MMV688766 has MIC80 values of 12.5–50 mM against various C. auris clades. 3) Supplementation with fatty acids reduced MMV688766's potency in C. auris and C. albicans. 4) MMV688766 induces lipid droplet accumulation without inhibiting fungal fatty acid synthase activity. 5) MMV688766 disrupts C. auris fatty acid homeostasis, depleting lipid levels without inhibiting FAS complex.
17	(Vargas‐Casanova et al., 2020)	Journal of Fungi	Columbia	Peptide **RWQWRWQWR** and an Ethanolic Extract of Bidens pilosa	1) Microplate reader 2) Antifungal Activity Assays 3) Time–Kill Curve 4) Checkerboard Assay 5) Cell Viability Assay 6) Scanning Electron Microscopy 7) Propidium Iodine Staining 8) Hemolytic Activity Assay 9) Cytotoxicity Assay	1) Peptide [5A]-R-1-R showed higher antifungal activity against C. auris than R-1-R. 2) B. pilosa extract combined with peptide enhanced antifungal activity against C. auris 3) Combining extract, peptide, and FLC enhanced antifungal effects and restored susceptibility in resistant strains. 4) The combination of peptide and extract enhanced antifungal activity, achieving FIC values of 0.12–0.68. 5) SEM revealed cell wall damage and increased permeability in C. auris treated with combinations.
18	(Perez-Castillo et al., 2021)	International Journal of Molecular Sciences	Brazil	**N-(4-Halobenzyl)**amides	1) Antifungal activity assays 2) Broth microdilution (BMD) antifungal susceptibility test	1) Compound 16 exhibited antifungal activity against Candida auris, with a MIC of 85.3 μg/mL.
19	Ramli et al. (2024)	Microbial Pathogenesis	Malaysia	2,3,4,4a-tetrahydro-1H-xanthen-1-one (**XA1**)	1) In vitro antifungal assay 2) Field emission scanning electron microscopy (FE-SEM) analysis 3) Microplate reading spectrophotometer 4) **P**ropidium iodide uptake assay 5) H2DCFDA assay 6) Biofilm inhibition assay 7) Stereomicroscopy 8) Fluorescence microscopy	1) Purified XA1 exhibited notable antifungal activity against Candida auris with 78.32 % inhibition at 100 μg/mL and a MIC of 50 μg/mL. 2) XA1-treated Candida auris cells showed significant surface damage, contraction, and roughness, especially at 2∗ MIC, compared to fluconazole. 3) The PI/FDA assay showed that XA1 treatment increases membrane permeability in Candida auris, evident by red PI staining and decreased green FDA fluorescence at MIC and 2∗MIC. 4) XA1 treatment increases ROS levels in Candida auris, boosting oxidative stress and inhibiting cell growth.
20	Torres et al. (2023)	Antibiotics	Columbia	**PNR20, PNR20**–**1,** and **35409**	1) Broth microdilution assay 2) Multiscan FC spectrophotometer 3) XTT reduction assay 4) Transmission Electron Microscopy 5) Flow Cytometry 6) In Vitro Cytotoxicity Assay	1) Peptide PNR20 displayed the broadest antifungal effect, inhibiting 50 % growth in all C. auris isolates. 2) Against C. auris H0059-13-1421, peptides PNR20, PNR20-1, and 35409 inhibited biofilm formation by 50 % after 24 and 72 h. 3) In C. auris H0059-13-1421 treated with peptides, organelle alterations occurred, with peptide 35409 causing small vacuole accumulation.
21	Vélez et al. (2024)	Cellular and Infection Microbiology	Columbia	C14R	1) Antifungal susceptibility testing 2) Antifungal synergism testing 3) Colorimetric methods 4) ELISA reader 5) C14R permeabilization assay	1) C14R showed strong activity against C. auris with MIC mode 6.25 μg/ml, ECV 95 % 25 μg/ml. 2) C14R and fluconazole showed synergy, reducing C14R MICs by 2–5 dilutions, fluconazole by 1–6, with FIC ≤0.5 μg/ml, achieving complete C. auris cell killing. 3) Treating C. auris biofilms with 25 μg/ml C14R reduced biofilm biomass and killed ∼90 % of cells. 4) C14R at 50 μg/ml slows C. auris growth for 48 h; 25 μg/ml is ineffective after 27 h. 5) In C. auris treated with C14R, membrane disruption and irregular cell shapes were observed.
22	Rather et al. (2022)	Journal of Fungi	Saudi Arabia	Human cathelicidin peptides LL-37	1) Broth microdilution assay 2) Cell Viability and Cell Count Assay 3) Antioxidant Assays 4) MuseTM Cell Analyzer 5) Propidium Iodide staining method 6) Fluorescence microscopy 7) Scanning Electron Microscopy	1) LL-37 showed potent activity against C. auris (MIC 25–100 μg/ml; MFC 3x MIC), with fungicidal effects. 2) LL-37 with FLZ, AmB, and CAS showed synergy against C. auris (FICI ≤0.5), reducing MICs 4–8-fold. 3) LL-37 reduced C. auris viability to 46.3 % at MIC and 15.1 % at MFC, showing potent inhibition. 4) LL-37 completely killed C. auris at MFC within 8 h and MIC within 24 h. 5) LL-37 increased CAT to 9.41 μmol/min at MIC; TBARS were 0.59 nmol at MIC, 1.1 nmol at MFC. 6) Cathelicidin LL-37 peptide induces cell cycle arrest in the S phase of C. auris. 7) LL-37 disrupted C. auris membrane integrity, increasing PI-positive cells, peaking at MFC concentrations. 8) LL-37 disrupted C. auris cell integrity, causing irregular shapes and surface depressions, indicating cell death.
23	Jaromin et al. (2024)	Antimicrobial Agents and Chemotherapy	Poland	PQA-Az-13	1) Skin colonization assay 2) MIC testing 3) Antifungal susceptibility testing 4) Mass Spectrometer 5) NanoDrop spectrophotometer 6) HPLC system 7) Thinfilm hydration method 8) Cryogenic transmission electron microscopy 9) Microplate reader 10) MTT assay	1) PQA-Az-13 showed strong antifungal activity against C. auris (MIC 0.67–1.25 μg/mL), outperforming fluconazole and amphotericin B. 2) PQA-Az-13 treatment increased extracellular vesicle size by 22 nm but decreased their concentration significantly in C. auris. **3)** PQA-Az-13 liposomes showed MIC values of 15.6–62.5 μg/mL, reducing C. auris biofilm by 83 %. 4) PQA-Az-13 inhibits C. auris biofilms, but its properties limit disinfectant applications and scalability.
24	Kubiczek et al. (2020)	Macromolecular Bioscience	Germany	**Cerberus** (Hydrogel)	1) Minimal inhibitory concentration assays 2) Broth microdilution method 3) Lambert–Pearson plot 4) Confocal laser scanning microscopy 5) Light microscopy 6) Miles and Misra method 7) Confocal laser scanning microscopy	1) YFP-LecB binds C. auris effectively in BSA/THPC hydrogel, achieving 90,000 cells mm^2^. 2) MIC for Cm-p5 against C. auris is 11 μg/mL; effective in human serum. 3) Cm-p5 released from gel reduced C. auris cells by 80 % at 9 μg/mL. 4) C. auris remained viable on collagen without hydrogel treatment, demonstrating infection risk. 5) C. auris cells near hydrogel showed impaired viability; direct contact eradicated them completely.
25	Vazquez-Munoz et al. (2020)	Antibiotics	USA	Bismuth nanoparticles (**BiNPs**)	1) Susceptibility Tests 2) Antibiofilm Activity Assays 3) Benchmark Microplate Reader 4) Scanning electron microscopy (SEM)	1) BiNPs exhibited strong anticandidal activity against C. auris strains, with MICs ranging from 1 to 4 μg/mL. 2) BiNPs inhibited C. auris biofilm formation with IC50 values ranging from 5.1 to 113.1 μg/mL. 3) BiNPs showed antibiofilm activity against C. auris with IC50 values lower than fluconazole (>64 μg/mL) but weaker than caspofungin (1–5 μg/mL). 4) Sublethal BiNP concentrations increased C. auris biofilm activity up to 2.5 times before declining at higher doses. 5) SEM analysis used BiNP concentrations near IC50 values to observe C. auris biofilm structure and morphology. 6) SEM revealed BiNP treatment reduced C. auris biofilm coverage and pseudohyphae presence at 16–128 μg/mL. 7) At 64 μg/mL, BiNPs showed minimal reduction in biofilm and no morphological changes in strain AR no. 0381. 8) BiNPs inhibited biofilm in strain AR no. 0383 but not in no. 0384; both showed altered morphology. 9) BiNPs at 32 μg/mL reduced biofilm in strains AR no. 0385 and no. 0386, altering yeast morphology.
26	Adnan et al. (2023)	Pharmaceutics	Hungary	**Anidulafungin** and **Micafungin** with and without Nikkomycin Z	1) Whole Genome Sequencing 2) MIC assays 3) Time–Kill Studies 4) One-way ANOVA	1) Anidulafungin and micafungin MICs were below CDC breakpoints, except isolate 28. Nikkomycin Z MICs ranged from 2 to >16 mg/L; 5/15 not inhibited. 2) Nikkomycin Z showed no significant CFU reduction; maximum decrease was 0.17 log CFU for isolate 20 (MIC 2 mg/L). 3) Anidulafungin showed a fungistatic effect, with highest CFU decrease of 1.4 log and k = 0.22 1/h k = 0.221/h for isolate 27 at 8–32 mg/L. 4) For isolate 28, nikkomycin Z plus anidulafungin showed transient CFU decrease 5) Anidulafungin at 32 mg/L achieved 3 log CFU decrease (T99.9 = 3.04 h); with nikkomycin Z, T99.9 reduced to 1.7–2.9 h. 6) Anidulafungin had weak effects against the South African clade; 8 mg/L nikkomycin Z increased activity and showed synergy with isolate 204 at 32 mg/L.
27	Gowri et al. (2020)	Journal of Applied Microbiology	India	Sertraline	1) ELISA reader 2) Time kill assay 3) PAFE 4) Inverted microscope 5) SEM 6) PI staining 7) flow cytometry 8) Sorbitol protection assay 9) Ergosterol effect assay 10) UV–Vis spectrophotometer	1) Sertraline's MIC against C. auris isolates: 20 μg/ml for 70, 40 μg/ml for 33, IL. 2) Sertraline achieved complete C. auris killing in 6 h, compared to 4 h with nystatin. 3) Sertraline induced a 4 h PAFE on C. auris, while nystatin showed a 5 h effect. 4) Sertraline (20 μg/ml) and nystatin inhibited C. auris hyphae formation, maintaining yeast form only. 5) Sertraline inhibited C. auris biofilm by 71 %, while nystatin showed 68 % biofilm reduction 6) Sertraline (20 μg/ml, 3 h) caused C. auris cell shrinkage and damage, similar to nystatin effects. **7)** Sertraline (20 μg/ml, 4 h) caused 55 % PI uptake in C. auris, versus 25 % with nystatin. Untreated cells showed none. 8) Sertraline's MIC remained unchanged with sorbitol, indicating no cell wall damage in C. auris. 9) Sertraline's MIC was unchanged with external ergosterol, indicating no direct ergosterol binding in C. auris. 10) Sertraline (11.5 μg) reduced C. auris ergosterol by 5.5-fold, while nystatin reduced it by 1.21-fold.
28	(Vargas‐Casanova et al., 2020)	Medicinal Chemistry & Drug Discovery	Colombia	LfcinB (21–25)Pal	1) MALDI-TOF MS 2) RP-HPLC 3) Broth microdilution method 4) Time kill assay 5) Checkerboard method	1) The palindromic peptide LfcinB (21–25)Pal demonstrated varying antifungal activity against C. auris, with MIC/MFC values of 100 μg/mL (67 μM). This activity is notable compared to other sequences. 2) LfcinB (21–25)Pal showed antifungal activity against C. auris (MIC 100 μg/mL), promising for treatment. 3) LfcinB (21–25)Pal showed fungicidal activity against C. auris 537 at 0.5 MIC for 72 h. 4) LfcinB (21–25)Pal showed fungistatic at 135 μM and fungicidal at 270 μM against C. auris. 5) LfcinB (21–25)Pal showed indifference with fluconazole (FIC index 1) against C. auris 537.
29	Ziental et al. (2022)	Pharmaceutics	Poland	Zinc (II) and Palladium (II) Phthalocyanine complexes	1) Susceptibility to PACT post-habituation 2) Light-Dependent Activity assay 3) Dark toxicity study 4) MIC tests 5) Antimicrobial activity assay	1) The palladium (II) Pc 4 showed strong photocytotoxicity against C. auris, with a high singlet oxygen quantum yield and enhanced efficacy. 2) Pc derivative 3 achieved >5.05 log reduction in C. auris at 100 μM and 100 J/cm^2^. 3) C. auris shows increased sensitivity to PACT, possibly due to high-efflux pump activity and metabolic strain.
30	Kamli et al. (2021)	Journal of Fungi	Saudi Arabia	Ag-Cu-Co trimetallic nanoparticles	1) Biological Assays 2) Broth microdilution assay 3) Cell count and viability assay 4) JC-10 mitochondrial membrane potential assay kit 5) SpectraMax iD3 multi-mode microplate reader 6) Apoptosis Detection Kit I 7) Flow Cytometer 8) MuseTM Cell Analyzer	1) The Ag-Cu-Co trimetallic nanoparticle demonstrated potent anti-Candida activity with MIC values of 0.39–0.78 μg/mL and MFC values of 0.78–1.56 μg/mL against Candida auris strains, outperforming fluconazole and comparable to caspofungin. 2) Ag-Cu-Co nanoparticles reduced C. auris viability to 52.4 %, 18.9 %, and 1.9 % at ½ MIC, MIC, and MFC. 3) Ag-Cu-Co nanoparticles induced Mitochondrial Membrane Potential depolarization in C. auris, with JC-10 ratios at ½ MIC: 1.53, MIC: 1.13, and MFC: 0.92. 4) Ag-Cu-Co nanoparticles in C. auris increased cytosolic Cytochrome *c* (1.23) and reduced mitochondrial Cytochrome *c* (0.78) at MFC. 5) Ag-Cu-Co nanoparticles induced PS externalization in C. auris, with apoptotic cells increasing at MIC and MFC. 6) Ag-Cu-Co nanoparticles caused C. auris G2/M arrest, with 48.8 % at MIC and 60.4 % at MFC. 7) Ag-Cu-Co nanoparticles showed minimal hemolysis in C. auris tests, with 0.63 % at MFC and 11.73 % at 3.12 μg/mL.
31	Eldesouky et al. (2018)	International Journal of Antimicrobial Agents	USA	Sulfamethoxazole and Azole antifungal drugs	1) Antifungal susceptibility testing 2) Chequerboard assay 3) Time–kill assay 4) Caenorhabditis elegans infection study 5) SpectraMax i3x microplate reader	1) Fluconazole effective in strains 381/387 (MIC 4 μg/mL); 5-fluorocytosine effective (0.25–1 μg/mL). 2) Sulfamethoxazole restored fluconazole susceptibility in strain 382 (FICI 0.156) and showed synergy with voriconazole in strains 382 (FICI 0.09), 383 (0.375), and 390 (0.50). 3) Sulfamethoxazole (64 μg/mL) and voriconazole (0.25 μg/mL) combined showed fungistatic effect in strain 382, similar to 5-fluorocytosine. 4) Combination of sulfamethoxazole (128 μg/mL) and voriconazole (0.5 μg/mL) increased C. elegans survival by ∼70 % over 5 days. 5) Sulfamethoxazole (128 μg/mL) did not inhibit MFS or ABC efflux pump activity, similar to glucose. 6) PABA supplementation (100 μg/mL) reversed sulfamethoxazole–voriconazole synergy in C. auris strain 382, increasing FICI to 0.75. 7) Sulfamethoxazole–fluconazole combination suppressed growth in azole-resistant mutants 11A8A2A and 10B1A3A, but not in efflux-activated strains SCMRR1R34A and SCTAC1R34A.
32	Oliveira et al. (2023)	Journal of Medicinal Chemistry	Brazil	Lead 2-Thiazolylhydrazones	1) In vitro growth inhibitory assay 2) In Vitro Antifungal Screening Using Broth Microdilution Assay 3) Assessment of Cell Viability by the MTT Assay 4) In Vitro Caco-2 Permeability Assay 5) High-performance liquid chromatography (HPLC) method	1) Compounds 17, 18, 19, and 22 showed potent antifungal activity against C. auris (320–780 nM MIC). 2) Compound 28 showed strong binding to C. auris FKS1 with a binding energy of −8.7 kcal/mol.
33	Kavaliauskas et al. (2024)	Antibiotics	Lithuania	3-((4-hydroxyphenyl)amino)propanoic acid derivatives 2–37	1) Minimal inhibitory concentration determination 2) Manual microplate viewer	1) The indolinone compounds 18 and 19 showed no antifungal or antibacterial activity (MIC >64 μg/mL) against C. auris. 2) Hydrazone derivatives **15 and 16** showed promising antifungal activity (MIC 8–16 μg/mL) against C. auris.
34	Reda and Fayed, 2023	Journal of the Australian Ceramic Society	Egypt	**calcium-doped zinc oxide** (ZC) ceramic nanoparticles	1) Scanning electron microscopy analysis (SEM) 2) Antimicrobial activity assay 3) Microplate reader	1) The MIC50 values for C. auris are: ZC0: 40.93 μg/ml, ZC10: 77.51 μg/ml, ZC90: 44.76 μg/ml (CDC-B11903); ZC0: 110.8 μg/ml, ZC10: 167.6 μg/ml, ZC90: 101.9 μg/ml (CDC-CAU9). 2) ZnO nanoparticles effectively disrupt multiple biomolecular pathways in C. auris, combatting resistance mechanisms.
35	(Chopra et al., 2020)	Drugs of the Future	India	Rezafungin acetate	1) Quantitative real-time PCR (qPCR) 2) Matrix-assisted laser desorption/ionization (MALDI) mass spectrometry 3) In vivo assay	1) Rezafungin shows potent in vitro activity against Candida auris, with MIC values ≤ 0.25 μg/mL, comparable to anidulafungin. 2) Rezafungin significantly reduces C. auris biofilm thickness, crucial for treating MDR infections. 3) Rezafungin reduced C. auris CFU by 1.04–3.85 log10 CFU/g in murine model.
36	Kamli et al. (2021)	Antioxidants	Saudi Arabia	Ag–Fe bimetallic NPs	1) Biological Assays 2) Antifungal susceptibility testing 3) Agar Diffusion Assay 4) Cell count and viability assay 5) JC-10 mitochondrial membrane potential assay kit 6) Microplate readers 7) Apoptosis Kit 8) MuseTM Cell Analyzer 9) Antioxidant Enzymes Assays 10) Thiobarbituric acid reactive substances (TBARS) method 11) Cytotoxicity Assay	1) Agglomerated Ag–Fe nanoparticles (∼14.30 ± 2.20 nm) show high cellular uptake and potent antifungal effects against C. auris. 2) Ag–Fe NPs showed strong antifungal activity against C. auris, with MIC 0.19–0.39 μg/mL and MFC 0.39–0.78 μg/mL. 3) Ag–Fe NPs showed strong antifungal activity against C. auris with ZOIs of 17–22 mm, while 1 % DMSO showed no effect. 4) Ag–Fe NPs reduced C. auris cell viability to 42.6 % at 1/2 MIC, 16.7 % at MIC, and 5.9 % at MFC. 5) Ag–Fe NPs disrupt mitochondrial membrane potential in C. auris, triggering apoptosis through mitochondrial depolarization and cytochrome *c* release. 6) Ag–Fe NPs induced apoptosis in C. auris, with 80.3 % in Q2 at 0.39 μg/mL, showing late apoptotic markers. 7) Ag–Fe NPs induced G2/M cell cycle arrest in C. auris, with 69.9 % in G2/M at 0.78 μg/mL 8) Ag–Fe NPs induced oxidative stress in C. auris, with catalase increasing 33.5-fold and TBARS 2.19-fold at MFC.
37	Xie et al. (2024)	Journal of Medicinal Chemistry	China	2,4,6- Trisubstituted Triazine Hydrazone Derivatives	1) In Vitro Antifungal Susceptibility Test 2) Time−Growth Curve Assay 3) Time−Kill Curve Assay 4) Biofilm Formation and Disruption Assay 5) Real-Time RT-PCR Assay 6) Cytotoxicity Assay 7) Hemolysis Assay	1) Hydroxyl triazine hydrazones 26 and 28 showed moderate activity (MIC 4.0, 2.0 μg/mL) against FCZ-resistant C. auris. 2) Triazine hydrazone compounds 26 and 28 showed broad antifungal activity against C. auris (MIC 0.5–16.0 μg/mL). 3) Morpholino-substituted triazine hydrazones (e.g., compound 41) showed reduced activity against C. auris (MIC >64.0 μg/mL).
38	(Kolge et al., 2023)	International Journal of Biological Macromolecules	India	C-PLGA nanoparticles	1) Biotek Synergy HT plate reader 2) Antifungal assays 3) In-vitro cytotoxicity and hemolysis 4) SEM analysis	1) C-PLGA-FLZ-NPs reduced C. auris MIC from 160 μg/ml (FLZ) to 2.5 μg/ml, a 64-fold decrease. 2) C-PLGA-FLZ-NPs reduced C. auris dye efflux from 77 % to 47 %, enhancing dye retention. 3) C-PLGA-FLZ-NPs reduced C. auris CFU to 9300, BUN to 11–15 mg/dl, and creatinine to 0.6 mg/dl
39	Negi et al. (2024)	Biomedicines (MDPI)	India	Graphene–silver nanocomposite	1) Cell Surface Hydrophobicity Assay 2) Agar-Well Diffusion Assay 3) Microbroth Dilution Assay 4) Quantitative Biofilm Inhibition Assays 5) Field Emission Scanning Electron Microscopy 6) MTT assay	1) C. auris strains showed varying hydrophobicity: NCCPF 470200 (80.7 %), NCCPF 470197 (36.4 %), NCCPF 4710203 (37.4 %). 2) GO-AgNPs showed significant antimicrobial activity against C. auris with notable inhibition zones. 3) GO-AgNPs inhibited C. auris biofilm formation by approximately 96 % at MIC and sub-MIC values. 4) GO-AgNP-functionalized catheters reduced C. auris adhesion and biofilm formation significantly.
40	(Al-Trawneh et al., 2020)	Chemical Papers	Jordan	Pyrazolo [5,1-c][1,2,4]triazine derivatives 3a–g	1) Broth microdilution method 2) Kinetic microplate reader	1) Compound 3d showed moderate antifungal activity against Candida auris, with MIC values of 16–64 μg/mL.
41	Eldesouky et al. (2020)	Scientific Reports	USA	Ospemifene	1) Standard broth microdilution checkerboard assays 2) Spectrophotometer 3) Nile red efux assay 4) Microplate reader 5) Caenorhabditis elegans assay	1) Ospemifene showed broad-spectrum synergy with itraconazole, reducing MIC by 2- to 64-fold (ΣFICI = 0.05–0.5) for most isolates, excluding C. glabrata; specific reductions were also observed for C. auris. 2) The ospemifene-itraconazole combination significantly inhibited growth of C. auris 390, reducing growth more than either drug alone. 3) In the C. elegans model, ospemifene-itraconazole reduced C. auris 390 CFU by 96 %, versus 71 % for itraconazole alone.
42	Bertašiut et al. (2023)	International Journal of Molecular Sciences	Lithuania	1-(2-Hydroxyphenyl)- and (3,5-Dichloro-2- hydroxyphenyl)-5-oxopyrrolidine-3-carboxylic Acid Derivatives	1) Standard broth microdilution methods 2) MTT assay 3) Microplate reader	1) Compound 15 exhibited antifungal activity (MIC 16 μg/mL) against three multidrug-resistant C. auris lineages. 2) Only compounds 26b and 27b showed antifungal activity against C. auris AR-381 (MIC 128 μg/mL).
43	Wani et al. (2024)	Royal Society of Chemistry	Saudi Arabia	Triazole bridged quinoline derivatives	1) Assessment of the minimum inhibitory concentration value 2) Broth microdilution assay 3) Assessment of the minimum fungicidal concentration value 4) Crystal violet assay **5)** Confocal microscopy 6) Microplate reader 7) Apoptosis Detection Kit	1) QT7 exhibited excellent activity against C. auris (MIC: 0.12 mg/mL, MFC: 0.24 mg/mL). 2) QT7 inhibited mature C. auris biofilm by 53.6 % at MIC, 81.98 % at MFC, and 89.57 % at 2 × MFC. 3) QT7 reduces mitochondrial membrane potential in C. auris, causing cytochrome *c* leakage and apoptosis. 4) QT7 induced dose-dependent apoptosis in C. auris, while H2O2 caused necrosis (Q1: 37.9 %). 5) QT7 (MIC: 0.12 mg/mL, MFC: 0.24 mg/mL) outperforms structurally similar derivatives against C. auris. 6) QT7 induces apoptosis in C. auris via phosphatidylserine exposure and mitochondrial depolarization.
44	Wani et al. (2023)	Bioorganic Chemistry	Saudi Arabia	pyrrolidine-based 1,2,3-triazole derivatives	1) Broth microdilution assay 2) MuseTM Count and Viability assay kit 3) JC-10 mitochondrial membrane potential assay kit–microplate 4) Microplate reader 5) Cytochrome *c* oxidase assay kit 6) Cytotoxicity assay	1) New pyrrolidine-based triazole P6 shows potent antifungal activity against C. auris (MIC: 0.977 μg/mL; MFC: 1.95 μg/mL). 2) P6 reduced C. auris viability dose-dependently: 39.4 % at 1/2 MIC, 20.9 % at MIC, 0.1 % at MFC. 3) P6 induced C. auris cell cycle arrest, with cells shifting to S-phase: 58.3 % at MIC, 62.2 % at 2∗MIC. 4) P6 reduced mitochondrial membrane potential in C. auris: JC-10 ratios were 0.82 (1/2 MIC), 0.59 (MIC), 0.15 (MFC). 5) P6 increased cytosolic cytochrome *c* in C. auris: values at MFC were 1.25 (cytosol), 0.46 (mitochondria).
45	Spadari et al. (2022)	Journal of Medical Mycology	Brazil	Ketoconazole/calix [n]arenes-based compounds	1) Antifungal susceptibility testing 2) Broth microdilution technique 3) Toxicity and antifungal efficacy in Galleria mellonella larval model	1) KTZ/calix [n]arenes inhibited C. auris at 0.03−0.12 mg/mL (low) and 0.25−16 mg/mL (high). 2) PM-CX6Na/KTZ reduced C. auris burden in Galleria mellonella at 20 mg/kg, enhancing survival.
46	Ni et al. (2023)	European Journal of Medicinal Chemistry	China	(2R,3R)-3-((3- substitutied-phenyl-isoxazol-5-yl)methoxy)-2-(2,4-difluorophenyl)-1-(1H-tetrazol-1-yl)butan-2-ol derivatives (**10a-10u**)	1) In vitro antifungal activities assay 2) Cell viability assay	1) Compound 10h showed outstanding antifungal activity against C. auris 922 with MIC ≤0.008 μg/mL.
47	Ramzan et al. (2022)	European Journal of Medicinal Chemistry	India	β-Nitrostyrene derivatives (SS45, SS46 and SS47)	1) Broth microdilution assay 2) Serial dilution spot assay 3) Checkerboard assay 4) Drug transport assays 5) Time kill assay 6) Scanning Electron Microscopy 7) Glycerol estimation.	1) The β-Nitrostyrene derivatives SS45, SS46, and SS47 exhibited potent antifungal activity against drug-resistant Candida auris strains, with MIC80 values of 2–4 μg/mL. 2) SEM images revealed that β-Nitrostyrene derivatives SS46 and SS47 caused significant cell surface deformation in C. auris after 3 h, with notable lysis at 6 h. 3) SS47 treatment significantly increased intracellular glycerol levels in other strain of candida, but less in C. auris. 4) SS46 and SS47 significantly increased ROS generation in C. auris, validated by fluorescence intensity measurements. 5) SS47 combined with cell wall agents showed marginal synergy against C. auris, FICI values were unchanged.

### 3.1 Cell viability and drug synergism

**Fig. 3:**
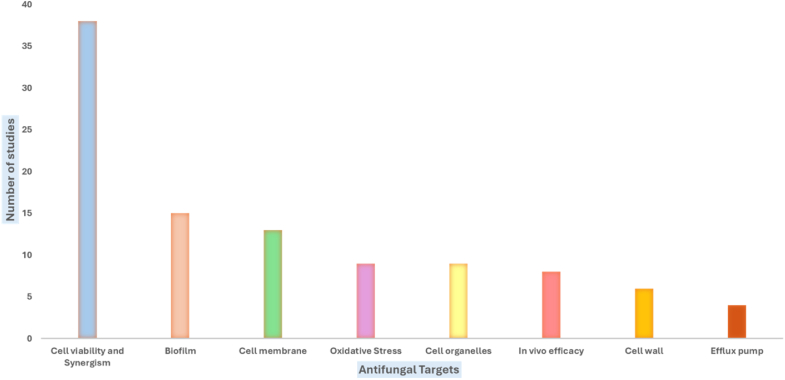
Distribution of antifungal targets of SCs reported in the included studies (n = 47) for *C. auris.*

Recent studies have shown that SCs, via drug synergism, have promising potential to reduce cell viability, particularly against fluconazole-resistant *C. auris*. The category of cell viability and synergism has attracted the most research, with a total of 38 studies (Fig. 3). This indicates that researchers have been focusing extensively on understanding how cells survive and how combining treatments can be more effective in combating fungal infections. SCs can be divided into three sub-categories, based on their chemical nature: i) small molecules, ii) peptides and peptide-based compounds and iii) nanomolecules and nanomaterials.

#### 3.1.1 Small molecules

In pharmaceutical development, a small molecule is defined as a chemically synthesized molecular entity with a low molecular weight of approximately 500 Da ideally, but generally less than 1000 Da (Reynolds et al., 2013). This definition is also used by the Food and Drug Administration (FDA) and the National Institutes of Health (NIH) to define small molecule drugs. The described SCs belong to this category (Table 2). Small molecules can be classified by their chemical structures, even if several SCs could be affiliated to different sub-class due to their particular structure which combined different chemical moieties. However, seven main sub-classes of small molecules have been retained in this review: i) haloaromatics derivatives, ii) hydrazone derivatives, iii) nitrogen-containing heterocycles, iv) echinocandins, v) olefins, vi) polycyclic compounds and vii) miscellaneous ones.

**Table 2:** Chemical categorization and structure of the SCs reported in the included studies.

SERIES		Entry	Structure	Reference
**HALOAROMATIC DERIVATIVES**		**1**	C4	Fatima et al. (2024)
		**2**	C13	
		**3**	CA	(Perez-Castillo et al., 2021)
		**4**	MYC-053	Tetz et al. (2019)
		**5**	Sertraline	Gowri et al. (2020)
**HYDRAZONE DERIVATIVES**		**6**	XI	Xie et al. (2024)
		**7**		Oliveira et al. (2023)
		**8**		Kavaliauskas et al. (2024)
		**9**		Bertašiut et al. (2023)
**AZO-HETEROCYCLES**	**THIAZOLE DERIVATIVES**	**10**	MO	Mohammad et al. (2019)
		**11**	MA1	Malukaite et al. (2021)
		**12**	MA2	
	**IMIDAZOLIUM**	**13**	NAIMS	Lee et al. (2021)
	**TRIAZOLE DERIVATIVES**	**14**	L-173	Levshin et al. (2024)
		**15**	pta derivatives	Srivastava et al. (2020)
		**16**	P6	Wani et al. (2023)
		**17**	QT7	Wani et al. (2024)
	**ISOXAZOLE**	**18**	MMV688766	Puumala et al. (2022)
	**TETRAZOLE**	**19**	NI	Ni et al. (2023)
	**TRIAZINE**	**20**	TR	(Al-Trawneh et al., 2020)
	**INDAZOLES**	**21**		Bertašiut et al. (2023)
		**22**	PQA-Az-13	Jaromin et al. (2024)
	**TALOCYANINE METALLIC COMPLEX**	**23**		Ziental et al. (2022)
**OTHERS**		**24**	XA1	Ramli et al. (2024)
		**25**	C14EtOH	Hafidi et al. (2022)
**ECHINOCANDINS**		**26**		Adnan et al. (2023)
		**27**		
**OLEFINS (polyènes, stilbenes …)**		**28**		Ramzan et al. (2022)
		**29**	Ospemifene	Eldesouky et al. (2020)
		**30**		Gowri et al. (2020)
**POLYCYCLES**		**31**	Ceragenin CSA-131 (R= C_12_H_25_)	Hashemi et al. (2018)
		**32**	SCY-247	Chu et al. (2021)
**PEPTIDES**		**33**	Peptide PNR20 was obtained by random selection of 10 polar and 10 nonpolar amino acids. Its sequence is not reported but the characteristics of this 20-mer peptide are their high content of helical structures, positive charge, and content of hydrophobic amino acids.	Torres et al. (2023)
		**34**	Palindromic peptide LfcinB (21–25) **RWQWRWQWR**	(Vargas‐Casanova et al., 2020)
		**35**	Fba **HHH-GVIYGKDVKDLF DYAQE**	Huang et al. (2024)
		**36**	Met6 **HHH-GFPRIGGQRELKKITEA**	Huang et al. (2024)
		**37**	C14R **CSSGSLWRLIRRFLRR**	Vélez et al. (2024)
		**38**	LL37 **LLGDFFRKSKEKIGKEFKRIVQRIKDFLRNLVPRTES**	Rather et al. (2022)
**COMPLEXE MOLECULES**		**39**	Mixture of PLA and HA, giving PLA-HA which is then submitted to electrospunning PLA + HA	Liu et al. (2022a)
		**40**	COP1T-HA	Liu et al. (2022b)
		**41**		Amann et al. (2023) Kubiczek et al. (2020)
		**42**		Spadari et al. (2022)

##### 3.1.1.1 Haloaromatic compounds

Among the haloaromatic compounds tested, carbazate derivatives C4 (Table 2, entry 1) and C13 (Table 2, entry 2) exhibited a marked decrease in cell viability. Other thiazole-based compounds (entries 11–12) showed selective antifungal activity. The *para*-chlorophenyl moiety in these derivatives was identified as crucial for targeting *C. auris* (Fatima et al., 2024; Malūkaitė et al., 2021). Interestingly, this moiety is also present in MYC-053, a promising agent that displayed potent inhibitory activity against *C. auris*, with precise half-maximal inhibitory concentration (IC_50_) (1 μg/ml) and minimum inhibitory concentration (MIC) (4 μg/ml) values (Entry 4, Tetz et al., 2019). Compound CA (Table 2, entry 3) demonstrated significant antifungal activity with an MIC of 85.3 μg/mL (Perez-Castillo et al., 2021). Repurposed drugs have also shown considerable potential. For example, sertraline (Table 2, Entry 5) eradicated *C. auris* completely in 6 h, a result comparable to that of nystatin, used as a reference control in the same study, (Table 2, Entry 26), which achieved completed cell death within 4 h (Gowri et al., 2020). Notably, sertraline also displayed a chlorinated aromatic moiety. This halogenated aromatic part, even if not mentioned as responsible for the measured activity, is also observed in other antifungal agents, belonging to different chemical sub-classes, such as compound XI (Table 2, entry 6, bearing a fluorinated one), thiazole derivative MA2 (Table 2, entry 12), triazole L-173 (Table 2, entry 14), MMV688766 (Table 2, entry 18), indazoles 29a-b (Table 2, entry 21) or PQA-Az-13 (Table 2, entry 22). This electro-attracting aromatic moiety appears to be importance. In some reported antifungals, the halogen atom is replaced by another strongly electro-attracting group such as a nitro substituent (Table 2, entries 8, 9 and 17).

##### 3.1.1.2 Hydrazone-based compounds

With regard to the hydrazone derivatives, the triazine hydrazone derivatives XI (see Table 2, entry 6) exhibited broad-spectrum antifungal activity, with MIC values ranging from 0.5 to 16.0 μg/mL (Xie et al., 2024). Small molecules have shown significant promise as antifungal agents. The thiazolylhydrazone series (Table 2, entry 7) exhibited potent activity against *C. auris*, with MIC values ranging from 320 to 780 nM (Oliveira et al., 2023). Hydrazone derivatives bearing a nitro five-membered heterocyclic ring (Table 2, Entry 8) demonstrated MIC values of 8–16 μg/mL, suggesting their potential as effective antifungal agents (Kavaliauskas et al., 2024). 5-Oxopyrrolidine-3-carboxylic acid Derivatives (Table 2, entry 9) were reported by Bertašiūtė et al., 2023 showing promising antifungal activity against three multidrug-resistant *Candida auris* lineages with an MIC of 16 μg/mL.

##### 3.1.1.3 Nitrogen-containing heterocycles

Many nitrogen-containing heterocycles have been reported as effective antifungals (Table 2, entries 10–23). A notable example is the synthetic phenylthiazole derivative compound MO, a, which demonstrated significant inhibition against eight resistant strains of *C. auris* at a concentration of 2 μg/ml. The effects were comparable to those of the widely used antifungal amphotericin B (Mohammad et al., 2019). Compounds MA1 and MA2, which also contain a thiazole moiety, were reported by Malukaite et al. (2021). Compound 7c from the NAIMS series (Table 2, Entry 13), a naphthalenyl-acyl-imidazolium salt, demonstrated significant antifungal activity against *Candida auris,* with a reported MIC value of 3.125 μg/ml, while Compound L-173, a triazole one (Table 2, Entry 14) surpassed voriconazole's efficacy, showing MIC values of 2 mg/ml compared to voriconazole's 8 mg/ml (Lee et al., 2021; Levshin et al., 2024). The same research group has reported three series of bridged triazole (Table 2, entries 15–17). Piperidine derivatives such as pta1, pta2, and pta3 (Table 2, entry 15) have been shown to have antimycotic efficacy against *C. auris*, with dose-dependent reductions in cell viability observed (Srivastava et al., 2020). Pyrrolidine-based triazole compounds (Table 2, entry 16) have been screened. Notably, when R = *para*-nitrophenyl, the compound exhibited a dose-dependent reduction in *C. auris* viability, achieving 39.4 % inhibition at 1/2 MIC, 20.9 % at MIC, and complete loss of viability at MFC (Wani et al., 2023). It should be noted that this series is structurally very similar to the pta derivatives reported by the same research group (Table 2, entry 16). In these compounds, the triazole ring is indeed bridged with a pyrrolidine moiety whereas in the pta-series, the additional moiety is a piperidine one. This change from a 5-membered ring to a 6- membered ring did not greatly impact the activity against Candida species. Wani et al. also reported the potency of QT7 (Table 2, entry 17) which has a quinoline aromatic moiety instead of an aliphatic cyclic amine. As will be shown later, this modification had a significant impact on the mode of action of the QT-series.

MMV688766, an isoxazole derivative, and Compound NI, a tetrazole featuring an isoxazole moiety have both demonstrated antifungal activity against Candida species (Table 2, entries 18–19). The trisubstituted isoxazole compound MMV688766 was found to be fungicidal, reducing *C. auris* growth by 80 % at a concentration of 25 mM (Puumala et al., 2022). Exceptional activity was demonstrated by compound NI, which achieved a MIC of ≤0.008 μg/mL against *C. auris* strain 922 (Ni et al., 2023).

Pyrazolo [5,1-c][1,2,4]triazine derivatives TR (Table 2, entry 20) exhibited moderate antifungal activity against *Candida auris* (MIC: 16–64 μg/mL), highlighting their potential in antifungal research (Al-Trawneh et al., 2020). By contrast, only indazole compounds 29a and 29b were effective against *C. auris* AR-381, albeit with a higher MIC of 128 μg/mL (Bertašiūtė et al., 2023). A novel indazole-based compound, PQA-Az-13 (Entry 22), outperformed standard antifungal agents such as fluconazole and amphotericin B, with MIC values ranging from 0.67 to 1.25 μg/mL (Jaromin et al., 2024). Similarly, Zn-phtalocyanine complex Pc derivative (Entry 23) achieved a >5.05 log reduction in *C. auris* populations at 100 μM with an energy dose of 100 J/cm^2^ (Ziental et al., 2022).

##### 3.1.1.4 Echinocandins

Echinocandins have also been used to fight against *Candida* infections. With a death rate constant (k) of 0.22 1/h for isolate 27 at concentrations of 8–32 mg/L, the echinocandin anidulafungin (Table 2, Entry 26) demonstrated the greatest reduction in colony-forming units (CFU) by 1.4 log and demonstrated fungistatic effects (Adnan et al., 2023). Furthermore, Rezafungin acetate (Table 2, Entry 27) reduced fungal burdens by 1.04–3.85 log10 CFU/g in a murine model, underscoring its clinical relevance (Chopra et al., 2020).

##### 3.1.1.5 Olefins

Olefins are also represented as antifungals. β-Nitrostyrene derivatives SS45, SS46, and SS47 (Entry 28) exhibited potent effects against drug-resistant *C. auris* strains, with 2–4 μg/mL MIC80 values. Although SS47 showed minimal synergy with cell wall-targeting agents, its efficacy as a standalone treatment highlights its potential (Ramzan et al., 2022). Combination therapies also show promise. Ospemifene (Table 2, Entry 29), which is also a stilbene derivative, was found to exhibit synergy with itraconazole. This reduced the MICs by between 2- to 64-fold across most isolates, including *C. auris*, with ΣFICI values ranging from 0.05 to 0.50 (Eldesouky et al., 2020). Nystatin, a commercially available polyene antifungal, also belongs to this sub-class (Table 2, entry 30).

##### 3.1.1.6 Polycyclic compounds

Two polycyclic molecules were also recently highlighted as antifungal agents (Table 2, Entries 31–32). In particular, SCY-247 (Table 2, Entry 31) demonstrated robust antifungal efficacy, with time-kill analysis indicating strong action against *C. auris* (Chu et al., 2021).

##### 3.1.1.7 Others

Nitrogen-based heterocycles are widely represented but other simple compounds also displayed good activity. Purified 2,3,4,4a-tetrahydro-1H-xanthen-1-one XA1 (Entry 24) showed promising results, achieving 78.32 % growth inhibition at 100 μg/mL and an MIC of 50 μg/mL (Ramli et al., 2024). The cationic surfactant C14EtOH (Entry 25) exhibited lower MIC values against *C. auris* compared to its counterparts, C14PrOH and C16PrOH, highlighting its enhanced efficacy (Hafidi et al., 2022).

#### 3.1.2 Peptides and peptide-based compounds

The peptide PNR20 (Table 2, entry 33) exhibited broad-spectrum antifungal activity, effectively inhibiting 50 % of the growth of all the *C. auris* isolates tested (Torres et al., 2023). The palindromic peptide LfcinB (21–25) (Table 2, entry 34) also displayed antifungal potential, with minimum inhibitory and fungicidal concentrations (MIC/MFC) of 100 μg/mL (67 μM). It exhibited fungicidal effects against *C. auris* at 0.5 MIC over 72 h, as well as fungistatic and fungicidal activities at concentrations of 135 μM and 270 μM, respectively (Vargas‐Casanova et al., 2020).

#### 3.1.3 Nanomolecules and nanomaterials

Nanoparticles have emerged as an effective means of combating *C. auris*. Kamli et al. found that Ag-Cu-Co nanoparticles reduced viability to 52.4 %, 18.9 %, and 1.9 % at 1/2 MIC, MIC, and MFC, respectively (Kamli et al., 2021a). Another study highlighted the remarkable antifungal properties of Ag-Fe nanoparticles, with MIC values of 0.19–0.39 μg/mL and MFCs of 0.39–0.78 μg/mL. They also produced inhibition zones of 17–22 mm and reduced cell viability to 42.6 % at 1/2 MIC, 16.7 % at MIC, and 5.9 % at MFC (Kamli et al., 2021b). The same study found that Ag-Fe nanoparticles showed potent antifungal properties, achieving MIC and MFC values of 0.19–0.39 μg/mL and 0.39–0.78 μg/mL, respectively, with inhibition zones of 17–22 mm and significant reductions in cell viability (Kamli et al., 2021b). Biogenic bismuth nanoparticles (BiNPs) have also demonstrated potent antifungal activity, with MIC values spanning from 1 to 4 μg/mL across various *C. auris* strains (Vazquez-Munoz et al., 2020).

A more advanced approach, involving PLA-HA, an antimicrobial photodynamic therapy (aPDT) nanofibrous membrane, demonstrated a 77 % reduction in cell viability upon illumination, revealing the potential for synergistic *C. auris* inhibition (Liu et al., 2022a) (Table 2, entry 39)

**Fig. 4:**
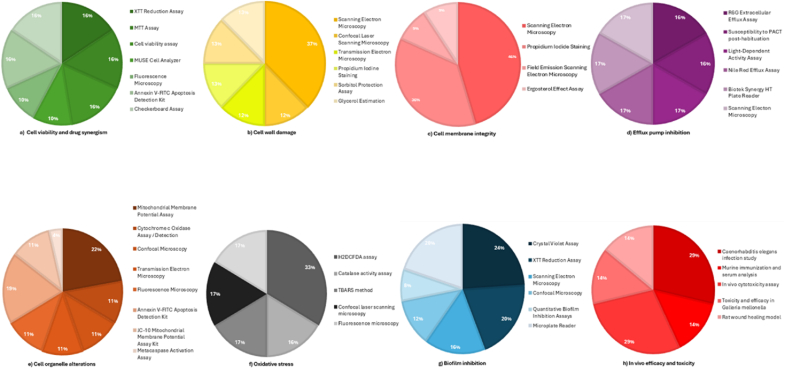
Distribution of detection methods used for analysing each mechanism of action for SCs in *C. auris.*

Nanoparticle-based delivery systems, such as C-PLGA-FLZ-NPs, which are composed of fluconazole loaded onto PLGA nanoparticles, reduced fungal colony-forming units (CFUs) to 9300 while maintaining low toxicity, as evidenced by stable blood urea nitrogen (BUN) levels of 11–15 mg/dL and creatinine levels of 0.6 mg/dL (Kolge et al., 2023). The most used methods, each accounting for 16 % of the included studies, are the XTT Reduction Assay, MTT Assay, Cell Viability Assay, Annexin V-FITC Apoptosis Detection Kit, and the Checkerboard Assay (Fig. 4). These methods are widely used to assess cell health and treatment interactions. Integrating SCs, nanoparticles, and small molecules has demonstrated significant potential for overcoming resistance and enhancing antifungal therapy against *C. auris*. Ongoing research into combinatorial strategies and novel molecular designs suggests that a breakthrough in *C. auris* management is imminent (Shbibe, 2023; Garza-Cervantes, 2021; Wong, 2013).

In conclusion, novel SCs are important in the fight against Candida infections, but other approaches are also promising. For instance, studies investigating drug synergy have also shown promise. Sulfamethoxazole restored fluconazole sensitivity in strain 382 (FICI - 0.156) and exhibited synergistic effects with voriconazole across multiple strains. However, supplementation with *p*-aminobenzoic acid (PABA) reversed this synergy in strain 382, increasing the FICI to 0.75 (Eldesouky et al., 2018).

### 3.2 Cell wall damage

The structural integrity of the cell wall is crucial for the survival and pathogenicity of *C. auris*. Recent studies have shed light on the factors that compromise this essential barrier, offering potential avenues for therapeutic intervention. Six studies have examined the cell wall, highlighting its importance as a target for antifungal strategies (Fig. 3). The most commonly used method for studying cell wall damage was Scanning Electron Microscopy analysis, accounting for 37 % of the included studies (Fig. 4). Fatima et al. (2024) highlighted disruptions to cell wall components, alongside alterations to vacuole morphology and acidification within *C. auris* cells. This process is critical for maintaining cell wall functionality. Treatment with PLA-HA (Table 2, entry 39) caused visible damage to the *C. auris* cell wall, as evidenced by the presence of red fluorescence, which contrasted sharply with intact walls observed in untreated controls (Liu et al., 2022a). Building on this, Liu et al., 2022a, Liu et al., 2022b showed that light-activated PLA-HA and Peptide RWQWRWQWRHA significantly damaged the *C. auris* cell wall while remaining relatively non-toxic to L929 mammalian cells, demonstrating its biocompatibility. Additionally, laser-activated COP1T-HA (Table 2, entry 40) was shown to effectively suppress *C. auris* growth, resulting in mild, observable damage to the cell wall. Scanning electron microscopy analysis corroborated these findings, revealing pronounced structural disruptions and increased wall vulnerability in cells treated with PLA-HA, COPIT-HA, and combinations involving *Bidens pilosa* extract (Liu et al., 2022a; Liu et al., 2022b; Casanova et al., 2023). The antifungal drug sertraline (Table 2, Entry 5) has also been investigated for its potential to disrupt *C. auris* cell wall. When applied at a concentration of 20 μg/ml for 3 h, sertraline (Table 2, entry 5) induced cell shrinkage and damage. However, the stability of its MIC in the presence of sorbitol suggests that the primary mechanism of action of sertraline does not involve direct interference with cell wall integrity (Gowri et al., 2020). Meanwhile, Ramzan et al. (2022) examined the synergistic potential of SS47 (Table 2, Entry 28) when combined with cell wall-targeting agents but found that there was no significant enhancement as the fractional inhibitory concentration index (FICI) values remained unchanged. While this lack of synergy indicates there is no additive effect against the cell wall, it does not provide direct evidence about SS47's mechanism of action. Dedicated biochemical or molecular studies would be required for this. The survival and pathogenicity of *C. auris* depend on maintaining the integrity of the cell wall. Research (Fatima et al., 2024; Liu et al., 2022a, Liu et al., 2022b; Casanova et al., 2023) highlights numerous interesting strategies that take advantage of flaws in this framework. Cutting-edge drugs like PLA-HA and COP1T-HA show great promise in attacking the cell wall, paving the way for new antifungal treatments to combat this dangerous pathogen's resistance (Liu et al., 2022a, Liu et al., 2022b; Casanova et al., 2023).

### 3.3 Cell membrane integrity disruption

Targeting the cell membrane is a key approach to combating *C. auris*, a multidrug-resistant fungal pathogen. Thirteen studies (Fig. 3) have focused on the cell membrane, highlighting efforts to understand how fungal cell membranes can be targeted to create more effective antifungal treatments. Maintaining the integrity of the plasma membrane is vital for cellular homeostasis, and disruption to it often triggers fungal cell death. Recent research has identified a variety of SCs that destabilize the membrane, impair its function, and induce downstream effector pathways. Fig. 4 illustrates the distribution of the various methods employed to study cell membrane integrity. SEM accounts for the largest proportion (46 %), demonstrating its pivotal role in examining the membrane's structure. PI staining follows with 36 %, highlighting its use in checking membrane permeability. The remaining 9 % is split between Field Emission Scanning Electron Microscopy and the ergosterol effect assay, which are used to understand changes in the membrane and how ergosterol impacts its function.

Several small molecules have been shown to compromise membrane integrity. For example, compounds such as C4 (Table 2, Entry 1) and C13 (Table 2, entry 2) disrupt membrane stability by depleting ergosterol, a key component of fungal membranes. This compromises the structural integrity of *C. auris* cells (Fatima et al., 2024). Similarly, the pta series of compounds (pta1, pta2, and pta3) (Table 2, entry 15) affect the plasma membrane, leading to destabilization and apoptosis-associated effects. Of these compounds, pta3 has been found to have a particularly pronounced impact, inducing phosphatidylserine (PS) externalization and DNA damage, especially at higher concentrations (Srivastava et al., 2020). Antimicrobial peptides and related agents also show significant promise. Ceragenin CSA-131 (Table 2, Entry 31) disrupts the *C. auris* cell membrane, causing significant morphological changes at higher concentrations (Hashemi et al., 2018). Similarly, human cathelicidin peptide LL-37 not only disrupts membrane integrity but also induces surface depressions and irregular shapes in fungal cells. This leads to increased uptake of propidium iodide (PI), indicating loss of viability. Notably, LL-37's activity peaks at minimum fungicidal concentrations (MFCs), highlighting its potency (Rather et al., 2022). In addition to directly disrupting the cell membrane, some compounds affect lipid homeostasis. MMV688766 (Table 2, entry 18), for example, promotes lipid droplet accumulation in *C. auris* cells while reducing overall lipid levels. Interestingly, this occurs without directly inhibiting fatty acid synthase, suggesting an alternative mechanism for its antifungal activity (Puumala et al., 2022). Similarly, β-nitrostyrene derivatives (e.g., SS47; Table 2, entry 28) elevate intracellular glycerol levels in other Candida species, albeit with a reduced effect in *C. auris* (Ramzan et al., 2022).

Combinatorial treatments have also been shown to comprise membrane integrity. For example, a combination of peptide [5A]-R-1-R and Bidens Pilosa extract was found to increase cell permeability when examined under an electron microscope, revealing structural damage in *C. auris* cells (Casanova et al., 2023). This approach highlights the potential of leveraging synergistic mechanisms to enhance antifungal activity. The emergence of novel antifungal agents also highlights the importance of membrane-targeting strategies. For example, triazole-bridged quinoline derivatives (e.g., QT7, see Table 2, entry 17) induce apoptosis in *C. auris* through PS exposure (Wani et al., 2024), while compounds like XA1 (Table 2, Entry 24) lead to surface contraction and roughness, particularly at concentrations above the MIC. Membrane permeability assays further confirm the activity of XA1 activity, showing reduced viability and increased PI staining (Ramli et al., 2024). Metal-based nanoparticles have also gained attention for their antifungal effects. Ag-Cu-Co nanoparticles, for example, disrupt the *C. auris* membrane by inducing PS externalization and increasing apoptotic markers at both MIC and MFC (Kamli et al., 2021b, Kamli et al., 2021a). This mode of action, coupled with their physical properties, makes nanoparticles a compelling area of study.

Lastly, the mechanism of action of certain existing antifungal agents has been revisited in the context of *C. auris*. For instance, sertraline (Table 2, entry 5) has been shown to significantly reduce ergosterol levels in *C. auris* despite not binding directly to it. This is evident from its unaltered MIC in the presence of exogenous ergosterol (Gowri et al., 2020). This contrasts with nystatin (Table 2, etry 30), which directly targets ergosterol but achieves a comparatively modest reduction. Disrupting the plasma membrane of *C. auris* through physical damage, metabolic interference or apoptosis enhances the efficacy of antifungals by destabilizing defences and triggering downstream effects, thereby highlighting the critical role of this mechanism in antifungal strategies.

### 3.4 Efflux pump inhibition

Efflux pumps play a key role in the development of drug resistance development in *C. auris*, which complicates antifungal therapy. Inhibiting these transporters is a promising approach to restoring drug susceptibility and combating multidrug resistance. However, with only four studies dedicated to efflux pumps, this area received the least research focus despite being one of the major mechanisms of drug resistance (Fig. 3). The R6G Extracellular Efflux Assay and the Nile Red Efflux Assay account for 16 % and 17 % of efflux studies, respectively (Fig. 4). Fatima et al. (2024) identified two carbazate derivatives, C4 (butyl 2-(4-chlorophenyl) hydrazine-1-carboxylate) (Table 2, Entry 1) and C13 (phenyl 2-(4-chlorophenyl) hydrazine-1-carboxylate) (Table 2, Entry 2), as potent inhibitors of ATP-binding cassette (ABC) transporters in *C. auris*. Their findings suggested that such small molecules could disrupt efflux mechanisms, thereby enhancing antifungal efficacy. Ziental et al. (2022) then demonstrated that coupling Pc derivative 3 with photodynamic antimicrobial chemotherapy (PACT) at 100 J/cm^2^ reduced the viability of *C. auris* by more than 5.05 log at 100 μM (Table 2, entry 23). Interestingly, *C. auris's* dependence on efflux pumps and the metabolic strain that ensues were linked to its increased susceptibility to PACT, rendering the pathogen more vulnerable to photodynamic damage. In contrast, sulfamethoxazole (128 μg/mL) did not inhibit ABC or major facilitator superfamily (MFS) transporters, behaving similarly to glucose as an inert efflux modulator. However, Eldesouky et al. (2018) reported that combining sulfamethoxazole with fluconazole suppressed the growth of azole-resistant mutants (11A8A2A and 10B1A3A). Nevertheless, this combination had no effect on strains with activated efflux mechanisms, such as SCMRR1R34A and SCTAC1R34A. These findings emphasize the importance of efflux-mediated resistance in specific *C. auris* phenotypes.

Emerging nanotechnology-based interventions have also shown promise in overcoming efflux-mediated resistance. For example, Kolge et al. (2022) developed chitosan-poly (lactic-co-glycolic acid) fluconazole nanoparticles (C-PLGA-FLZ-NPs), which reduced the minimum inhibitory concentration (MIC) of fluconazole against *C. auris* from 160 μg/mL to 2.5 μg/mL, a remarkable 64-fold reduction. The nanoparticles also significantly decreased dye efflux from 77 % to 47 %, indicating substantial inhibition of efflux activity and enhanced intracellular drug retention. Efflux pump inhibitors are a promising approach to supporting antifungal therapies against *C. auris*. These can be chemical agents or nanotechnological techniques. Further study in this area may reveal practical ways to deal with multidrug resistance in this difficult pathogen.

### 3.5 Cell organelle alterations

Understanding the mechanisms driving antifungal resistance and apoptosis in *C. auris* requires an appreciation of alterations in cell organelles, particularly mitochondria and vacuoles Nine studies have investigated cell organelles, likely exploring how structures such as mitochondria are affected by antifungal treatments (Fig. 3). The Mitochondrial Membrane Potential assay is used in most of the studies, accounting for 22 % of the analysis of alternations to cell organelle. This method reveals changes in mitochondrial function and their impacts on cells (Fig. 4). These organelle changes shed light on potential therapeutic targets for combating this multidrug-resistant pathogen. SCs such as C4 and C13 (Table 2, Entries 1–2) disrupt vacuolar morphology acidification, a critical process for cellular homeostasis. This disruption also impairs the growth of *C. auris* on non-fermentative carbon sources, suggesting that mitochondrial dysfunction may be a key mechanism involved (Fatima et al., 2024). Similarly, other molecules, including pta1, pta2, and pta3 (Table 2, Entry 15), induce mitochondrial depolarization, reducing membrane potential and triggering apoptosis through cytochrome C release. This subsequently activates caspases (Srivastava et al., 2020). Antifungal agents such as QT7 and P6 (Table 2, entries 16–17) have demonstrated strong mitochondrial-targeting effects. For example, QT7 induces apoptosis in *C. auris* by decreasing the mitochondrial membrane potential, causing cytochrome C leakage and activating downstream apoptotic pathways. It also exhibits activity against biofilm formation, an essential feature in fungal pathogenesis, further suggesting its therapeutic potential (Wani et al., 2023, 2024). P6 was also shown to significantly decrease mitochondrial membrane potential and increase cytosolic cytochrome C, emphasizing its capacity to induce programmed cell death Wani et al. (2023), (Table 2, entry 16). Reactive oxygen species (ROS)-mediated mitochondrial damage is another critical factor. For example, photoactivated PLA-HA treatment induces ROS production, leading to apoptosis through mitochondrial disruption (Liu et al., 2022a). Ag-Cu-Co and Ag-Fe nanoparticles have been found to depolarize mitochondrial membranes, thereby increasing the release of cytosolic cytochrome C and inducing apoptosis (Kamli et al., 2021a; Kamli et al., 2021b). These nanoparticles cause dose-dependent mitochondrial dysfunction, which reinforces their potential as antifungal agents.

Finally, organelle-specific effects on vacuoles and extracellular vesicles have been observed. Treatments with certain peptides, such as peptide 35409, lead to the accumulation of small vacuoles, while agents like PQA-Az-13 (Table 2, Entry 22) affect extracellular vesicle dynamics, reducing their concentration but increasing size. These changes could potentially impair fungal communication and pathogenesis (Torres et al., 2023; Jaromin et al., 2024). Studying these organelle alterations helps us to understand how *C. auris* reacts to antifungal treatments and reveals weaknesses in the pathogen. This knowledge is crucial for devising targeted strategies to address the issue directly.

### 3.6 Oxidative stress

The induction of oxidative stress is a well-studied mechanism for combating *C. auris*. Nine studies focus on oxidative stress and explore how fungal cells deal with reactive oxygen species (Fig. 3). Various agents utilise reactive oxygen species (ROS) to disrupt cellular processes and promote antifungal activity. In many studies, H2DCFDA is a commonly used agent in many studies, accounting for 33 % of oxidative stress analysis (Fig. 4). For instance, Fatima et al. (2024) reported that SCs C4 and C13 (Table 2, Entries 1–2) induce oxidative stress in *C. auris*, resulting in lipid peroxidation and DNA damage. These effects were reversible with antioxidant treatment. Lipid peroxidation was assessed using TBARS analysis, and reactive oxygen species (ROS) production was quantified through DCFDA staining. Similarly piperidine derivatives (Table 2, entry 15) have been shown to induce DNA damage, cell cycle arrest, and apoptosis in *C. auris* (Srivastava et al., 2020). Liu et al. (2022a) demonstrated that PLA-HA-based antimicrobial photodynamic therapy (aPDT) triggers apoptosis in *C. auris* through ROS production (Table 2, entry 39). XA1 (Table 2, Entry 24) treatment also elevates ROS levels, resulting in increased oxidative stress and inhibition of cell growth in *C. auris* (Ramli et al., 2024). P6 treatment shifts *C. auris* cells to the S-phase, with 58.3 % arrested at MIC and 62.2 % at twice the MIC Wani et al. (2023); (Table 2, entry 16). Similarly, SS46 and SS47 (Table 2, Entry 28) were found to significantly enhance ROS generation in *C. auris*, as validated by fluorescence intensity measurements (Ramzan et al., 2022).

In addition to these chemical agents, antimicrobial peptides and nanoparticles have demonstrated promising antifungal activity mediated by oxidative stress. The cathelicidin LL-37 peptide induces cell cycle arrest in the S-phase of *C. auris* (Rather et al., 2022, Table 2, entry 38), while Ag-Cu-Co nanoparticles cause G2/M cell cycle arrest, with 48.8 % of cells arrested at MIC and 60.4 % at MFC (Kamli et al., 2021a). Ag–Fe nanoparticles have proven particularly effective in inducing apoptosis in *C. auris* with late apoptotic markers evident in 80.3 % of cells in Q2 at a concentration of 0.39 μg/mL. They also cause significant G2/M cell cycle arrest, affecting 69.9 % of cells at a concentration of 0.78 μg/mL. Furthermore, these nanoparticles amplify oxidative stress, as demonstrated by a 33.5-fold increase in catalase activity and a 2.19-fold rise in TBARS levels at MFC (Kamli et al., 2021b). Taken together, these results emphasize the potential of targeting oxidative stress in developing effective treatments against *C. auris* and suggest new avenues for antifungal therapy.

### 3.7 Inhibition of biofilm formation

The ability of various agents to inhibit *C. auris* biofilms has been extensively studied, with promising results highlighting a variety of mechanisms of action. Of the included studies, fifteen focus on biofilms (Fig. 3). This indicates a significant interest in the study of biofilms, which are clumps of microbes that stick together, making fungal infections more difficult to treat. Compound 1 and amphotericin B were found to effectively inhibit *C. auris* biofilms at 1 × MIC, although efficacy diminished at sub-MIC concentrations. These compounds significantly reduced biofilm metabolic activity, with more pronounced effects observed at higher MICs (Mohammad et al., 2019). Similarly, SCs C4 and C13 substantially impaired biofilm formation, metabolic activity, biomass accumulation, and epithelial adherence (Fatima et al., 2024, Table 2, entries 1–2). Dimeric compounds, such as Dimer 1 and Dimer 2 (Table 2, entry 41), exhibited moderate planktonic activity, yet proved highly effective against biofilms, with IC_50_ values of 23.2 μg/mL and 33.3 μg/mL against strains M1 and M2, respectively (Amann et al., 2023). Innovative strategies, including the use of Cm-p5 dimers, showed significant inhibition of mature biofilms, achieving up to 95 % reduction in growth (Kubiczek et al., 2020). In comparison, caragenin CSA-131 (Table 2, entry 31) was able to penetrate the biofilms of *C. auris* and *C. albicans*, killing the cells without significantly altering the structure of the biofilms (Hashemi et al., 2018). Advanced photodynamic therapies, such as PLA-HA and COP1T-HA (Table 2, entries 39–40), effectively eradicated biofilms. PLA-HA reduced biofilm viability by 77 % upon illumination, whileCOP1T-HA induced substantial structural damage and cell lysis (Liu et al., 2022a; Liu et al., 2022b). Peptides including PNR20, PNR20-1, and 35409 (Table 2, entry 33) inhibited biofilm formation by around 50 % after 24–72 h of treatment (Torres et al., 2023). At 25 μg/mL, C14R (Table 2, entry 37) reduced biofilm biomass and achieved ∼90 % cell killing, showcasing its potential (Vélez et al., 2024). Liposomal formulations such as PQA-Az-13 (Table 2, Entry 22) demonstrated MIC values of 15.6–62.5 μg/mL and reduced biofilm formation by 83 %. However, its scalability for widespread applications requires further investigation (Jaromin et al., 2024). BiNPs (bismuth nanoparticles) inhibited biofilm formation with IC_50_ values ranging from 5.1 to 113.1 μg/mL, demonstrating stronger activity than fluconazole but weaker than caspofungin. Morphological analyses revealed reduced biofilm coverage and alterations in yeast morphology in some strains, though some isolates displayed limited susceptibility (Vazquez-Munoz et al., 2020). Sertraline and nystatin effectively maintained the yeast form of *C. auris*, with sertraline reducing biofilm by 71 % and nystatin by 68 % (Gowri et al., 2020). Rezafungin acetate (Table 2, Entry 27) significantly reduced biofilm thickness, highlighting its potential for combating multidrug-resistant infections (Thakare et al., 2020). QT7 demonstrated dose-dependent efficacy, inhibiting mature biofilms by 53.6 % at MIC, 81.98 % at MFC, and 89.57 % at 2 × MFC (Wani et al., 2024, (Table 2, entry 17).

Another important factor affecting *C. auris's* ability to form biofilms is its hydrophobicity. The hydrophobicity of several strains, including NCCPF 470200 (80.7 %), NCCPF 470197 (36.4 %), and NCCPF 4710203 (37.4 %), varies significantly. This impacts adhesion characteristics and biofilm development. Agents suchs as GO-AgNPs exploit this property to greatly reduce adhesion and colonization on medical devices such as catheters by 96 % at MIC and sub-MIC levels (Negi et al., 2024). According to Reda and Fayed, 2023, zinc oxide (ZnO) nanoparticles have shown great promise in disrupting several biomolecular pathways in *Candida auris*, thereby combating its resistance mechanisms effectively. The Crystal Violet assay is commonly used in many studies for biofilm inhibition, accounting for 24 % of the analyses. Conversely, quantitative biofilm inhibition assays account for just 8 %, offering an alternative approach to studying biofilm formation (Fig. 4). These findings underscore the multifaceted approaches being developed to tackle *C. auris* biofilms, which are less susceptible to conventional antifungal agents, and demonstrate how these approaches can help combat this challenging pathogen in clinical settings.

### 3.8 In vivo efficacy and toxicity

A total of eight studies focus on *in vivo* efficacy, demonstrating an emphasis on testing the effectiveness of antifungal treatments in living organisms (Fig. 3). The *C. elegans* infection model and the *in vivo* cytotoxicity assay are both commonly used in many studies, accounting for 29 % of the analysis each (Fig. 4). These methods help to evaluate the effectiveness and potential toxicity of treatments in living organisms. According to Mohammad et al. (2019), compound MO (Table 2, Entry 10) has shown considerable effectiveness in prolonging the survival of nematodes infected with *C. auris*, performing at a level comparable to the antifungal agent 5-fluorocytosine. Similarly, Fatima et al. (2024) demonstrated that SCs C4 and C13 (Table 2, Entries 1–2) can enhance the survival of *Caenorhabditis elegans* infected with *C. auris*. These compounds demonstrated low haemolytic activity and minimal toxicity to the host, making them promising candidates for antifungal treatment with strong safety profiles. Huang et al. (2024) demonstrated that SNAP-based vaccines induced a robust IgG immune response and significantly improved survival rates in mice infected with *Candida auris*, outperforming the MP12 vaccine. Additionally, peptide-SNAP formulations provided enhanced protection, resulting in higher survival rates and substantial reductions in fungal burden. The effectiveness of these vaccines in promoting protective immunity was confirmed by the decreased number of kidney colony-forming units (CFUs) in mouse models of invasive candidiasis. PLA-HA conjugated with illumination exhibited potent antifungal activity against *C. auris*, resulting in significant cellular damage and minimal toxicity to L929 fibroblast cells. This approach demonstrated excellent biocompatibility. Furthermore, photodynamic therapy using PLA-HA accelerated wound healing in *C. auris*-infected rat models, minimizing inflammation and displaying minimal side effects (Liu et al., 2022a).

Ag-Cu-Co nanoparticles emerged as another promising intervention, demonstrating low haemolytic activity at effective concentrations against *C. auris*. Haemolysis was reported to be as low as 0.63 % at the minimum fungicidal concentration (MFC) and 11.73 % at 3.12 μg/mL, which supports their safety profile for potential therapeutic use (Kamli et al., 2021a). A combination of sulfamethoxazole (128 μg/mL) and voriconazole (0.5 μg/mL) increased the survival rate of *C. elegans* infected with *C. auris* by around 70 % over a five-days period (Eldesouky et al., 2018). Similarly, the combination of ospemifene and itraconazole reduced *C. auris* CFUs by 96 % in *C. elegans*, compared to a 71 % reduction with itraconazole alone (Eldesouky et al., 2020). Ketoconazole (KTZ) combined with calix [n]arenes (Table 2, entry 42) exhibited potent inhibitory effects against C. *auris* at low concentrations (0.03−0.12 mg/mL) with broader activity observed at higher concentrations (0.25−16 mg/mL). The PM-CX6Na/KTZ complex further demonstrated its therapeutic potential in a *Galleria mellonella* model by reducing the fungal burden and enhancing survival at a dosage of 20 mg/kg (Spadari et al., 2022). In summary, a diverse range of *in vivo* studies highlights the potential of novel compounds, nanoparticle formulations, combination therapies, and innovative vaccination strategies in combating *C. auris*. The balance of efficacy and minimal toxicity of these interventions is prominsing and underpins their significance in addressing the urgent need for effective antifungal treatments.

## 4 Conclusion and future perspectives

When it comes to treating *C. auris* infections, developing of innovative strategies and modifying drugs holds promise for more effective treatments that target specific pathogenic mechanisms. This review highlights SCs and advanced therapeutic approaches that can be refined to enhance their efficacy. These modifications can improve the penetration and activity, particularly in treating severe infections in sensitive organs such as the central nervous system (CNS), urinary tract (UTIs), bloodstream, and intra-abdominal areas – some of the most critical and potentially deadly sites affected by *C. auris* infections. Leveraging nanoparticle-based systems, including those with enhanced drug stability and bioavailability enables researchers to engineer targeted delivery methods. For instance, PLGA-functionalized nanoparticles or metal-based particles can be modified to maximize penetration and therapeutic action while minimizing systemic toxicity. This is particularly relevant for CNS infections, which are challenging due to the blood-brain barrier, and for deep-seated organ infections.

Compounds such as SCY-078 (ibrexafungerp) provide a useful point of comparison in this context. The broad activity of SCY-078 against resistant *C. auris*, combined with its oral bioavailability, makes it a valuable tool for treating systemic infections, including intra-abdominal infections and UTI cases. Conversely, SCY-247 shows promise in terms of potentially superior tissue penetration and efficacy, particularly for infections in sensitive sites such as the CNS. These findings highlight the importance of designing highly targeted, stable antifungal therapies that can effectively bypass physiological barriers.

Therapeutic design can also prioritize specific mechanisms, such as efflux pump inhibition or oxidative stress pathways. Tailoring drugs to disrupt biofilm formation on medical devices and within host tissues can address a critical resistance mechanism. For instance, combining antifungals with adjuvants that enhance biofilm penetration could improve outcomes in cases involving invasive devices such as catheters or ventilators. Future perspectives also include developing synthetic antimicrobial peptides and vaccine formulations, which could offer preventive benefits. SNAP-based peptide vaccines, for instance, can be optimized to elicit stronger immune responses, providing long-term immunity to high-risk populations. Overall, the discussed strategies suggest a way for tailor antifungal therapies to more effectively target *C. auris* in various clinical scenarios. However, global collaboration and rigorous clinical testing are essential to translate these laboratory findings into effective, widespread treatments.

## Author contributions

YS: search, data extraction, validation. YS and ZF: data analysis. ZF and SH: supervision. YS and SH: writing, original draft. MB: data analysis, review and editing of the manuscript. ZF and SH contributed to the conception and design of the study and review and editing of the manuscript. All the authors approved the final submitted version of the manuscript.

## Credit authors statement

Yamini Saini (YS), Zeeshan Fatima (ZF), Muriel Billamboz (MB) and Saif Hameed (SH)

YS: search, data extraction, validation. YS and ZF: data analysis. ZF and SH: supervision. YS and SH: writing, original draft. MB: data analysis, review and editing of the manuscript. ZF and SH contributed to the conception and design of the study and review and editing of the manuscript. All the authors approved the final submitted version of the manuscript.

## Funding

No specific funding has been received for this study.

## Declaration of competing interest

The authors declare that they have no known competing financial interests or personal relationships that could have appeared to influence the work reported in this paper.

## Data Availability

All the data related to the study is available within the manuscript.
